# *catena*-Poly[[dichloridozinc(II)]-μ-1,4-bis­(3-pyridylmeth­yl)piperazine]

**DOI:** 10.1107/S1600536809013877

**Published:** 2009-04-18

**Authors:** Aaron M. Hardy, Robert L. LaDuca

**Affiliations:** a Lyman Briggs College, Department of Chemistry, Michigan State University, East Lansing, MI 48825, USA

## Abstract

In the title compound, [ZnCl_2_(C_16_H_20_N_4_)]_*n*_, tetra­hedrally coordinated divalent Zn atoms are ligated by two Cl atoms and two N-donor atoms from two 1,4-bis­(3-pyridylmeth­yl)­piperazine (3-bpmp) ligands. The tethering 3-bpmp ligands promote the formation of [ZnCl_2_(3-bpmp)]_*n*_ chains situated parallel to (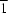
02). These chains aggregate *via* C—H⋯Cl inter­actions to form supra­molecular layers, which in turn stack to construct the three-dimensional crystal structure.

## Related literature

The structure was refined from a merohedrally twinned crystal; for the generation of reflection data from the major twin component, see: Sheldrick (2007 ▶). For 1,4-bis­(3-pyridylmeth­yl)piperazine coordination polymers of copper aryl­carboxyl­ates, see: Johnston *et al.* (2008 ▶). For the synthesis of the ligand, see: Pocic *et al.* (2005 ▶).
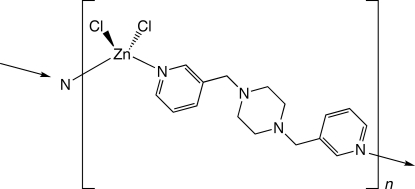


## Experimental

### 

#### Crystal data

[ZnCl_2_(C_16_H_20_N_4_)]
*M* *_r_* = 404.63
Monoclinic, 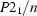

*a* = 11.4474 (4) Å
*b* = 13.0007 (4) Å
*c* = 12.4234 (4) Å
β = 95.909 (2)°
*V* = 1839.08 (10) Å^3^
*Z* = 4
Mo *K*α radiation
μ = 1.63 mm^−1^
*T* = 173 K
0.38 × 0.21 × 0.13 mm

#### Data collection

Bruker APEXII CCD area-detector diffractometer
Absorption correction: multi-scan (TWINABS; Sheldrick, 2007 ▶) *T*_min_ = 0.568, *T*_max_ = 0.813
21222 measured reflections
6035 independent reflections
3366 reflections with *I* > 2σ(*I*)
*R*_int_ = 0.059

#### Refinement

*R*[*F* ^2^ > 2σ(*F* ^2^)] = 0.047
*wR*(*F* ^2^) = 0.132
*S* = 1.10
6035 reflections
208 parameters
H-atom parameters constrained
Δρ_max_ = 0.58 e Å^−3^
Δρ_min_ = −0.60 e Å^−3^

### 

Data collection: *APEX2* (Bruker, 2006 ▶); cell refinement: *SAINT* (Bruker, 2006 ▶); data reduction: *SAINT*; program(s) used to solve structure: *SHELXS97* (Sheldrick, 2008 ▶); program(s) used to refine structure: *SHELXL97* (Sheldrick, 2008 ▶); molecular graphics: *CrystalMaker* (Palmer, 2007 ▶); software used to prepare material for publication: *SHELXL97*.

## Supplementary Material

Crystal structure: contains datablocks I, global. DOI: 10.1107/S1600536809013877/ng2573sup1.cif

Structure factors: contains datablocks I. DOI: 10.1107/S1600536809013877/ng2573Isup2.hkl

Additional supplementary materials:  crystallographic information; 3D view; checkCIF report

## Figures and Tables

**Table 1:** Hydrogen-bond geometry (Å, °)

*D*—H⋯*A*	*D*—H	H⋯*A*	*D*⋯*A*	*D*—H⋯*A*
C5—H5⋯Cl2^i^	0.95	2.77	3.718 (2)	176
C15—H15⋯Cl1^ii^	0.95	2.75	3.698 (2)	173

Symmetry codes: (i) 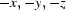; (ii) 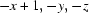.

**Fig. 1.**
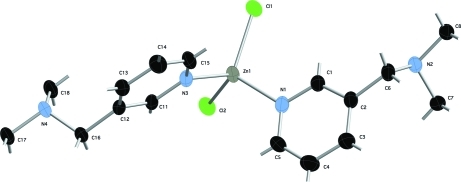
The asymmetric unit of the title compound, showing 50% probability ellipsoids and atom numbering scheme. Hydrogen atom positions are shown as sticks. Colour codes: gray Zn, green Cl, blue N, black C.

**Fig. 2.**
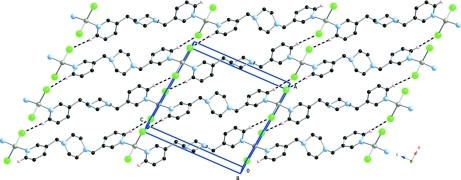
A layer of [ZnCl2(3-bpmp)]n chains in the title compound. C—H···Cl interactions are shown as dashed lines.

**Fig. 3.**
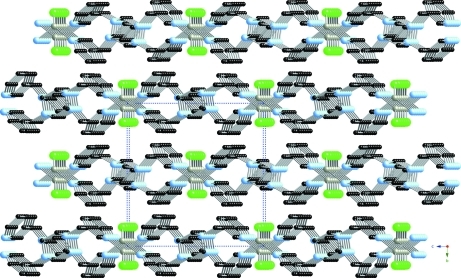
Stacking of layer motifs in the title compound.

## supplementary crystallographic information

### Comment

In comparison to coordination polymers based on the rigid rod tether 4,4'- bipyridine, extended solids based on the hydrogen-bonding capable bis(3-pyridylmethyl)piperazine (3-bpmp) ligand are much less common (Johnston *et al.*, 2008). The title compound was obtained during an attempt to prepare a zinc azide 3-bpmp coordination polymer.

The asymmetric unit of the title compound consists of a divalent Zn atom, two Cl atoms, and two halves of two crystallographically distinct 3-bpmp molecules. The coordination environment at Zn is a slightly distorted {ZnCl_2_N_2_} tetrahedron, with two chloro ligands and two N donor atoms from crystallographically distinct bis(3-pyridylmethyl)piperazine (3-bpmp) ligands (Figure 1).

Neighboring Zn atoms are bridged by tethering 3-bpmp ligands to construct neutral [ZnCl_2_(3-bpmp)]_n_ coordination polymer chains, that are oriented parallel to the (1 0 2) crystal direction. There are crystallographic inversion centres at the centroids of the piperazinyl rings within the 3-bpmp ligands. The through-ligand Zn···Zn distances within the chain motifs are 14.218 (4) and 14.259 (4) Å. These chains aggregate by C—H···Cl interactions to construct a supramolecular layer that is oriented parallel to the *ac* crystal planes (Figure 2). In turn these layer motifs stack by means of crystal packing forces to establish the three-dimensional crystal structure of the title compound (Figure 3).

### Experimental

Zinc chloride dihydrate and sodium azide were obtained commercially. Bis(3-pyridylmethyl)piperazine (3-bpmp) was prepared *via* a published procedure (Pocic *et al.*, 2005). Zinc chloride dihydrate (0.082 g, 0.48 mmol) was dissolved in 6 ml water in a glass vial. A 2 ml aliquot of tetrahydrofuran was carefully layered on the top of the zinc chloride solution. Above the tetrahydrofuran layer was gently placed a mixture of sodium azide (0.065 g, 1.0 mmol) and 3-bpmp (134 mg, 0.500 mmol) taken up in 5.5 ml of a 10:1 methanol:water mixture. Colourless blocks of the title compound deposited after standing at 25 °C for one week.

### Refinement

All H atoms bound to C atoms were placed in calculated positions, with C—H = 0.95 Å and refined in riding mode with *U*_iso_ = 1.2*U*_eq_(C).

### Crystal data

**Table:**

[ZnCl_2_(C_16_H_20_N_4_)]	*F*(000) = 832
*M**_r_* = 404.63	*D*_x_ = 1.461 Mg m^−^^3^
Monoclinic, *P*2_1_/*n*	Mo *K*α radiation, λ = 0.71073 Å
Hall symbol: -P 2yn	Cell parameters from 6035 reflections
*a* = 11.4474 (4) Å	θ = 2.3–32.2°
*b* = 13.0007 (4) Å	µ = 1.63 mm^−^^1^
*c* = 12.4234 (4) Å	*T* = 173 K
β = 95.909 (2)°	Block, colourless
*V* = 1839.08 (10) Å^3^	0.38 × 0.21 × 0.13 mm
*Z* = 4	

### Data collection

**Table:**

Bruker APEXII CCD area-detector diffractometer	6035 independent reflections
Radiation source: fine-focus sealed tube	3366 reflections with *I* > 2σ(*I*)
graphite	*R*_int_ = 0.059
ω–ψ scans	θ_max_ = 32.2°, θ_min_ = 2.3°
Absorption correction: multi-scan (TWINABS; Sheldrick, 2007)	*h* = −17→16
*T*_min_ = 0.568, *T*_max_ = 0.813	*k* = 0→19
21222 measured reflections	*l* = 0→17

### Refinement

**Table:**

Refinement on *F*^2^	Primary atom site location: structure-invariant direct methods
Least-squares matrix: full	Secondary atom site location: difference Fourier map
*R*[*F*^2^ > 2σ(*F*^2^)] = 0.047	Hydrogen site location: inferred from neighbouring sites
*wR*(*F*^2^) = 0.132	H-atom parameters constrained
*S* = 1.10	*w* = 1/[σ^2^(*F*_o_^2^) + (0.0539*P*)^2^ + 0.1746*P*] where *P* = (*F*_o_^2^ + 2*F*_c_^2^)/3
6035 reflections	(Δ/σ)_max_ < 0.001
208 parameters	Δρ_max_ = 0.58 e Å^−^^3^
0 restraints	Δρ_min_ = −0.60 e Å^−^^3^

### Special details

**Table:**

Experimental. Reflection data were collected on a non-merohedrally twinned crystal. The twin law was determined with *CELL*NOW (Sheldrick, 2003). The structure was solved and refined using reflections from only the major twin component, whose reflection file was generated using TWINABS (Sheldrick, 2007). Composite reflections belonging to both twin domains were omitted from the reflection list, causing the loss of 246 reflections from the major twin component data. The data set was still 99.9% complete to 2θ of 50°.
Geometry. All e.s.d.'s (except the e.s.d. in the dihedral angle between two l.s. planes) are estimated using the full covariance matrix. The cell e.s.d.'s are taken into account individually in the estimation of e.s.d.'s in distances, angles and torsion angles; correlations between e.s.d.'s in cell parameters are only used when they are defined by crystal symmetry. An approximate (isotropic) treatment of cell e.s.d.'s is used for estimating e.s.d.'s involving l.s. planes.
Refinement. Refinement of *F*^2^ against ALL reflections. The weighted *R*-factor *wR* and goodness of fit *S* are based on *F*^2^, conventional *R*-factors *R* are based on *F*, with *F* set to zero for negative *F*^2^. The threshold expression of *F*^2^ > σ(*F*^2^) is used only for calculating *R*-factors(gt) *etc*. and is not relevant to the choice of reflections for refinement. *R*-factors based on *F*^2^ are statistically about twice as large as those based on *F*, and *R*- factors based on ALL data will be even larger.

### Fractional atomic coordinates and isotropic or equivalent isotropic displacement parameters (Å^2^)

**Table:**

	*x*	*y*	*z*	*U*_iso_*/*U*_eq_	
Zn1	0.25226 (2)	0.03824 (2)	0.00021 (2)	0.02591 (10)	
Cl1	0.41393 (5)	0.13442 (5)	0.00653 (5)	0.03268 (16)	
Cl2	0.08858 (5)	0.13279 (5)	−0.00242 (5)	0.03181 (16)	
N1	0.25698 (18)	−0.06042 (15)	−0.12735 (16)	0.0271 (4)	
N2	0.48104 (17)	−0.04111 (15)	−0.39476 (16)	0.0268 (5)	
N3	0.24771 (17)	−0.05999 (15)	0.12836 (16)	0.0261 (4)	
N4	0.04009 (17)	−0.07296 (15)	0.42481 (16)	0.0252 (4)	
C1	0.3501 (2)	−0.05974 (18)	−0.18528 (19)	0.0266 (5)	
H1	0.4074	−0.0074	−0.1706	0.032*	
C2	0.3670 (2)	−0.13096 (18)	−0.26506 (19)	0.0262 (5)	
C3	0.2813 (2)	−0.2056 (2)	−0.2866 (2)	0.0344 (6)	
H3	0.2886	−0.2556	−0.3413	0.041*	
C4	0.1844 (2)	−0.2069 (2)	−0.2272 (2)	0.0401 (7)	
H4	0.1249	−0.2575	−0.2413	0.048*	
C5	0.1758 (2)	−0.13440 (19)	−0.1481 (2)	0.0331 (6)	
H5	0.1104	−0.1366	−0.1068	0.040*	
C6	0.4782 (2)	−0.12722 (18)	−0.31972 (19)	0.0284 (5)	
H6A	0.5461	−0.1218	−0.2638	0.034*	
H6B	0.4863	−0.1923	−0.3597	0.034*	
C7	0.3966 (2)	−0.05658 (19)	−0.49056 (19)	0.0293 (6)	
H7A	0.3164	−0.0624	−0.4680	0.035*	
H7B	0.4149	−0.1215	−0.5268	0.035*	
C8	0.5991 (2)	−0.03190 (19)	−0.4310 (2)	0.0292 (6)	
H8A	0.6196	−0.0966	−0.4666	0.035*	
H8B	0.6575	−0.0205	−0.3677	0.035*	
C11	0.1506 (2)	−0.06356 (18)	0.18122 (18)	0.0252 (5)	
H11	0.0869	−0.0193	0.1581	0.030*	
C12	0.1394 (2)	−0.12847 (17)	0.26723 (19)	0.0242 (5)	
C13	0.2323 (2)	−0.19399 (18)	0.3005 (2)	0.0284 (5)	
H13	0.2279	−0.2391	0.3601	0.034*	
C14	0.3317 (2)	−0.1922 (2)	0.2450 (2)	0.0337 (6)	
H14	0.3956	−0.2371	0.2654	0.040*	
C15	0.3364 (2)	−0.12444 (19)	0.1598 (2)	0.0311 (6)	
H15	0.4047	−0.1234	0.1223	0.037*	
C16	0.0267 (2)	−0.12896 (19)	0.32236 (19)	0.0271 (5)	
H16A	−0.0371	−0.0972	0.2736	0.032*	
H16B	0.0040	−0.2009	0.3358	0.032*	
C17	−0.0598 (2)	−0.09447 (19)	0.4855 (2)	0.0301 (6)	
H17A	−0.0649	−0.1694	0.4983	0.036*	
H17B	−0.1332	−0.0726	0.4425	0.036*	
C18	0.0478 (2)	0.03892 (18)	0.4078 (2)	0.0309 (6)	
H18A	−0.0237	0.0631	0.3634	0.037*	
H18B	0.1164	0.0545	0.3682	0.037*	

### Atomic displacement parameters (Å^2^)

**Table:**

	*U*^11^	*U*^22^	*U*^33^	*U*^12^	*U*^13^	*U*^23^
Zn1	0.02662 (16)	0.03010 (17)	0.02220 (16)	−0.00085 (13)	0.00820 (11)	0.00006 (13)
Cl1	0.0279 (3)	0.0348 (3)	0.0370 (4)	−0.0033 (2)	0.0115 (3)	−0.0063 (3)
Cl2	0.0271 (3)	0.0334 (3)	0.0361 (4)	0.0018 (2)	0.0088 (3)	0.0044 (3)
N1	0.0284 (11)	0.0316 (11)	0.0219 (10)	0.0012 (9)	0.0055 (8)	−0.0017 (9)
N2	0.0242 (10)	0.0342 (12)	0.0226 (11)	0.0022 (8)	0.0049 (8)	0.0021 (9)
N3	0.0262 (10)	0.0323 (11)	0.0206 (10)	0.0014 (8)	0.0062 (8)	−0.0006 (9)
N4	0.0259 (10)	0.0263 (10)	0.0252 (11)	−0.0019 (8)	0.0112 (8)	0.0008 (9)
C1	0.0290 (12)	0.0280 (13)	0.0232 (13)	−0.0012 (10)	0.0048 (10)	0.0008 (10)
C2	0.0261 (12)	0.0305 (13)	0.0225 (13)	0.0047 (10)	0.0046 (10)	0.0014 (10)
C3	0.0325 (14)	0.0361 (15)	0.0352 (15)	0.0007 (11)	0.0067 (12)	−0.0090 (12)
C4	0.0343 (15)	0.0385 (16)	0.0487 (18)	−0.0093 (12)	0.0100 (13)	−0.0132 (14)
C5	0.0253 (13)	0.0404 (15)	0.0348 (15)	−0.0037 (11)	0.0093 (11)	−0.0030 (12)
C6	0.0302 (13)	0.0336 (14)	0.0219 (13)	0.0029 (10)	0.0059 (10)	0.0014 (11)
C7	0.0255 (12)	0.0366 (14)	0.0254 (13)	−0.0027 (10)	0.0006 (10)	−0.0002 (11)
C8	0.0247 (12)	0.0383 (15)	0.0247 (13)	0.0002 (10)	0.0026 (10)	0.0016 (11)
C11	0.0236 (12)	0.0328 (13)	0.0196 (12)	0.0001 (10)	0.0045 (9)	−0.0017 (10)
C12	0.0257 (12)	0.0272 (12)	0.0205 (12)	−0.0013 (10)	0.0059 (10)	−0.0042 (10)
C13	0.0293 (13)	0.0315 (13)	0.0247 (13)	−0.0008 (10)	0.0048 (10)	0.0023 (11)
C14	0.0270 (13)	0.0382 (15)	0.0361 (15)	0.0078 (11)	0.0039 (11)	0.0042 (12)
C15	0.0258 (13)	0.0391 (15)	0.0301 (14)	0.0008 (11)	0.0117 (11)	−0.0005 (12)
C16	0.0243 (12)	0.0335 (14)	0.0247 (13)	−0.0057 (10)	0.0093 (10)	−0.0017 (11)
C17	0.0339 (13)	0.0275 (13)	0.0314 (14)	−0.0038 (10)	0.0158 (11)	−0.0007 (11)
C18	0.0367 (14)	0.0285 (14)	0.0299 (14)	−0.0033 (11)	0.0146 (11)	0.0032 (11)

### Geometric parameters (Å, °)

**Table:**

Zn1—N1	2.044 (2)	C6—H6B	0.9900
Zn1—N3	2.046 (2)	C7—C8^i^	1.511 (3)
Zn1—Cl1	2.2282 (6)	C7—H7A	0.9900
Zn1—Cl2	2.2383 (6)	C7—H7B	0.9900
N1—C5	1.344 (3)	C8—C7^i^	1.511 (3)
N1—C1	1.346 (3)	C8—H8A	0.9900
N2—C6	1.459 (3)	C8—H8B	0.9900
N2—C7	1.468 (3)	C11—C12	1.378 (3)
N2—C8	1.473 (3)	C11—H11	0.9500
N3—C15	1.343 (3)	C12—C13	1.392 (3)
N3—C11	1.349 (3)	C12—C16	1.522 (3)
N4—C17	1.460 (3)	C13—C14	1.389 (3)
N4—C16	1.461 (3)	C13—H13	0.9500
N4—C18	1.474 (3)	C14—C15	1.382 (3)
C1—C2	1.384 (3)	C14—H14	0.9500
C1—H1	0.9500	C15—H15	0.9500
C2—C3	1.386 (3)	C16—H16A	0.9900
C2—C6	1.505 (3)	C16—H16B	0.9900
C3—C4	1.394 (4)	C17—C18^ii^	1.503 (3)
C3—H3	0.9500	C17—H17A	0.9900
C4—C5	1.372 (4)	C17—H17B	0.9900
C4—H4	0.9500	C18—C17^ii^	1.503 (3)
C5—H5	0.9500	C18—H18A	0.9900
C6—H6A	0.9900	C18—H18B	0.9900
			
N1—Zn1—N3	102.50 (8)	N2—C7—H7B	109.5
N1—Zn1—Cl1	106.94 (6)	C8^i^—C7—H7B	109.5
N3—Zn1—Cl1	114.23 (6)	H7A—C7—H7B	108.1
N1—Zn1—Cl2	114.98 (6)	N2—C8—C7^i^	110.5 (2)
N3—Zn1—Cl2	105.42 (6)	N2—C8—H8A	109.5
Cl1—Zn1—Cl2	112.54 (2)	C7^i^—C8—H8A	109.5
C5—N1—C1	118.2 (2)	N2—C8—H8B	109.5
C5—N1—Zn1	121.65 (17)	C7^i^—C8—H8B	109.5
C1—N1—Zn1	119.74 (16)	H8A—C8—H8B	108.1
C6—N2—C7	110.91 (19)	N3—C11—C12	123.1 (2)
C6—N2—C8	109.81 (19)	N3—C11—H11	118.5
C7—N2—C8	108.15 (19)	C12—C11—H11	118.5
C15—N3—C11	118.2 (2)	C11—C12—C13	118.4 (2)
C15—N3—Zn1	122.47 (17)	C11—C12—C16	120.1 (2)
C11—N3—Zn1	119.25 (16)	C13—C12—C16	121.4 (2)
C17—N4—C16	109.71 (18)	C14—C13—C12	118.8 (2)
C17—N4—C18	108.93 (18)	C14—C13—H13	120.6
C16—N4—C18	111.69 (19)	C12—C13—H13	120.6
N1—C1—C2	123.7 (2)	C15—C14—C13	119.3 (2)
N1—C1—H1	118.1	C15—C14—H14	120.4
C2—C1—H1	118.1	C13—C14—H14	120.4
C1—C2—C3	117.3 (2)	N3—C15—C14	122.1 (2)
C1—C2—C6	119.2 (2)	N3—C15—H15	118.9
C3—C2—C6	123.4 (2)	C14—C15—H15	118.9
C2—C3—C4	119.5 (2)	N4—C16—C12	111.88 (19)
C2—C3—H3	120.3	N4—C16—H16A	109.2
C4—C3—H3	120.3	C12—C16—H16A	109.2
C5—C4—C3	119.4 (3)	N4—C16—H16B	109.2
C5—C4—H4	120.3	C12—C16—H16B	109.2
C3—C4—H4	120.3	H16A—C16—H16B	107.9
N1—C5—C4	122.0 (2)	N4—C17—C18^ii^	111.0 (2)
N1—C5—H5	119.0	N4—C17—H17A	109.4
C4—C5—H5	119.0	C18^ii^—C17—H17A	109.4
N2—C6—C2	112.85 (19)	N4—C17—H17B	109.4
N2—C6—H6A	109.0	C18^ii^—C17—H17B	109.4
C2—C6—H6A	109.0	H17A—C17—H17B	108.0
N2—C6—H6B	109.0	N4—C18—C17^ii^	110.45 (19)
C2—C6—H6B	109.0	N4—C18—H18A	109.6
H6A—C6—H6B	107.8	C17^ii^—C18—H18A	109.6
N2—C7—C8^i^	110.8 (2)	N4—C18—H18B	109.6
N2—C7—H7A	109.5	C17^ii^—C18—H18B	109.6
C8^i^—C7—H7A	109.5	H18A—C18—H18B	108.1
			
N3—Zn1—N1—C5	54.4 (2)	C1—C2—C6—N2	−73.3 (3)
Cl1—Zn1—N1—C5	174.89 (18)	C3—C2—C6—N2	109.9 (3)
Cl2—Zn1—N1—C5	−59.4 (2)	C6—N2—C7—C8^i^	−179.14 (19)
N3—Zn1—N1—C1	−117.99 (18)	C8—N2—C7—C8^i^	−58.7 (3)
Cl1—Zn1—N1—C1	2.48 (19)	C6—N2—C8—C7^i^	179.64 (19)
Cl2—Zn1—N1—C1	128.20 (16)	C7—N2—C8—C7^i^	58.5 (3)
N1—Zn1—N3—C15	63.97 (19)	C15—N3—C11—C12	1.5 (3)
Cl1—Zn1—N3—C15	−51.3 (2)	Zn1—N3—C11—C12	179.02 (17)
Cl2—Zn1—N3—C15	−175.38 (18)	N3—C11—C12—C13	−0.6 (4)
N1—Zn1—N3—C11	−113.45 (18)	N3—C11—C12—C16	−179.5 (2)
Cl1—Zn1—N3—C11	131.27 (16)	C11—C12—C13—C14	−0.8 (3)
Cl2—Zn1—N3—C11	7.20 (18)	C16—C12—C13—C14	178.1 (2)
C5—N1—C1—C2	0.0 (4)	C12—C13—C14—C15	1.1 (4)
Zn1—N1—C1—C2	172.65 (18)	C11—N3—C15—C14	−1.1 (4)
N1—C1—C2—C3	1.1 (4)	Zn1—N3—C15—C14	−178.54 (19)
N1—C1—C2—C6	−176.0 (2)	C13—C14—C15—N3	−0.2 (4)
C1—C2—C3—C4	−0.9 (4)	C17—N4—C16—C12	−167.5 (2)
C6—C2—C3—C4	176.0 (2)	C18—N4—C16—C12	71.6 (3)
C2—C3—C4—C5	−0.3 (4)	C11—C12—C16—N4	−102.5 (2)
C1—N1—C5—C4	−1.2 (4)	C13—C12—C16—N4	78.6 (3)
Zn1—N1—C5—C4	−173.8 (2)	C16—N4—C17—C18^ii^	179.2 (2)
C3—C4—C5—N1	1.4 (4)	C18—N4—C17—C18^ii^	−58.2 (3)
C7—N2—C6—C2	−70.0 (3)	C17—N4—C18—C17^ii^	57.9 (3)
C8—N2—C6—C2	170.5 (2)	C16—N4—C18—C17^ii^	179.2 (2)

Symmetry codes: (i) −*x*+1, −*y*, −*z*−1; (ii) −*x*, −*y*, −*z*+1.

### Hydrogen-bond geometry (Å, °)

**Table:**

*D*—H···*A*	*D*—H	H···*A*	*D*···*A*	*D*—H···*A*
C5—H5···Cl2^iii^	0.95	2.77	3.718 (2)	176
C15—H15···Cl1^iv^	0.95	2.75	3.698 (2)	173

Symmetry codes: (iii) −*x*, −*y*, −*z*; (iv) −*x*+1, −*y*, −*z*.
